# Atrial Fibrillation Ablation Without Fluoroscopy: Because We Can

**DOI:** 10.19102/icrm.2018.091103

**Published:** 2018-11-15

**Authors:** Rahul N. Doshi

**Affiliations:** ^1^Division of Cardiology, Keck School of Medicine, University of Southern California, Los Angeles, CA, USA

**Keywords:** Ablation, atrial fibrillation, fluoroscopy, 3D mapping

In this month’s issue of *The Journal of Innovations in Cardiac Rhythm Management,* Percell et al.^1^ report a case of atrial tachycardia after pulmonary vein isolation for atrial fibrillation (AF), in which ablation was performed twice, with both instances occurring without the use of fluoroscopy. Two different mapping systems were used for the two procedures. Given that one was successful (and thus one was not), the obvious question to consider is whether or not one system is superior versus the other when utilizing zero-fluoroscopy techniques for the catheter ablation of these arrhythmias.

The authors detail a case in which propagation maps from both systems reveal a tachycardia originating in the high posteroseptal right atrium (RA) or superior vena cava (SVC) antrum. The tachycardia mechanism is difficult to discern, as pacing maneuvers were not possible secondary to tachycardia termination. The SVC antrum is a known trigger for AF.^2^ Certainly, the location is characteristic for both automatic and microreentrant mechanisms, especially after right pulmonary venous isolation. We are not given any information regarding the arrhythmogenicity of the right superior pulmonary vein, which does appear to be associated with SVC triggers.^3^ Regardless, both maps reveal a similar location. Whether the successful ablation was a result of targeting a focal breakout that had shifted after prior ablation or whether an anatomic barrier to reentry was created is impossible to discern.

There is a growing amount of literature regarding the use of zero-fluoroscopy approaches utilizing three-dimensional mapping. These data are summarized in **Table 1**. The first report describing the technique was published in 2009 by Ferguson et al.,^4^ who evaluated 21 patients while employing the EnSite™ system (Abbott Laboratories, Chicago, IL, USA). This was soon followed by the completion of a study by Reddy et al.^5^ in 2010. These early investigations demonstrated the feasibility of performing a fluoroless technique in conjunction with electroanatomic mapping and intracardiac echocardiography. Meanwhile, Razminia et al.^6^ first reported the feasibility of a fluoroless approach with the cryoballoon.

Regarding more recent research, the authors of the present case previously reported in *The Journal of Innovations in Cardiac Rhythm Management* on their one-year, single-center experience with the ablation of AF using zero-fluoroscopy and the CARTO^®^ mapping system (Biosense Webster, Diamond Bar, CA, USA).^7^ In this study, they compared 20 patients treated using radiofrequency (RF) ablation and zero-fluoroscopy with 30 patients treated with traditional RF and fluoroscopy and 22 patients treated with cryoablation (52 patients total) and found no significant differences in the clinical outcomes, safety, or time of the procedure except for fluoroscopy exposure. Additionally, Bulava et al.^8^ reported on a small, randomized trial employing a similar technique performed using the CARTO^®^ mapping system (Biosense Webster, Diamond Bar, CA, USA) and intracardiac ultrasound to eliminate fluoroscopy completely. They randomized 80 patients in a 1:1 fashion and found no differences in safety or procedural outcomes. O’Brien et al.^9^ shared (after a 24-patient “development phase” followed by a 45-patient “implementation phase”) data from 55 patients evaluated using only electroanatomic mapping and transesophageal echocardiography. Razminia et al.^10^ detailed their five-year experience with 500 consecutive ablation cases including 186 patients with AF; of these, 156 were treated with traditional RF ablation and 30 patients were treated with the cryoballoon while utilizing both CARTO^®^ (Biosense Webster, Diamond Bar, CA, USA) and EnSite™ (Abbott Laboratories, Chicago, IL, USA) three-dimensional mapping. Based on these reports, in single-center experiences, zero-fluoroscopy techniques for AF ablation are largely feasible and appear to be safe and equally effective as compared with traditional ablation. In addition, it is important to note that there is a “learning curve,” as procedure times tend to decrease with increasing experience.^10^

The largest series to date was recently reported by Lyan et al.^11^ These authors retrospectively reviewed 481 consecutive patients with paroxysmal AF and compared the safety and efficacy between a nonfluoroscopic approach (n = 245) and a traditional approach (n = 236). The procedure time was 108.8 minutes ± 18.2 minutes in the zero-fluoroscopy group, with no differences in either procedural outcome or complication rate. Thus, evidence suggests that the zero-fluoroscopy approach can be employed safely without compromising procedure efficiency with either CARTO^®^ (Biosense Webster, Diamond Bar, CA, USA) or EnSite™ (Abbott Laboratories, Chicago, IL, USA) three-dimensional mapping. However, to the best of my knowledge, a multicenter, prospective, randomized trial either comparing a zero-fluoroscopy approach with conventional imaging or comparing different mapping systems with one another has not been reported yet.

It is well-established that complex electrophysiologic procedures such as AF ablation are traditionally associated with longer fluoroscopy times in comparison with other types of ablation procedures.^12,13^ It has also been demonstrated that the use of electroanatomic mapping can reduce the amount of fluoroscopy.^14,15^ Technology improvements that have been made in three-dimensional mapping systems, such as the introduction of the ability to incorporate fluoroscopic images, have also been shown to further decrease X-ray exposure.^16^ The MediGuide^®^ system (Abbott Laboratories, Chicago, IL, USA) is a nonfluoroscopic imaging modality that allows catheter manipulation to occur on a prerecorded X-ray image and that can be employed for AF ablation.^17^ Sommer et al.^18^ recently reported their experience of 1,000 patients treated with this near-zero fluoroscopic technique. The average procedure time was 120 minutes ± 40.4 minutes, with a median fluoroscopic duration of 0.90 minutes ± 2.7 minutes. Although these improvements in technology are seemingly effective, there are also simple measures that can be implemented that only require a change in procedural habits. Knecht et al.^19^ demonstrated that simply removing one’s lead apron after transseptal puncture and relying solely on electroanatomic mapping from that point on can have dramatic effects on X-ray exposure. Notably, the mean fluoroscopy time in their study for the treatment group was 4.2 minutes (range: 2.6–5.6 minutes).

Regardless of the approach, with increasing expertise and advancements in technology, fluoroscopy exposure is decreasing. Casella et al.^20^ recently shared the results of fluoroscopy exposure over a seven-year period from seven different operators. The authors revealed the existence of significant variability in X-ray exposure among these operators during AF ablation. However, all operators showed a significant decrease in fluoroscopy time during AF ablation over the seven-year period. For AF procedures in the authors’ laboratory, the average fluoroscopy time was 23 minutes (range: 15–35 minutes), which corresponds to an average dose area product of 7,373 cGy × cm^2^, an effective dose of 16.0 mSv, and a lifetime attributable risk of 0.16%. Thus, even with respect to this modern cohort, the authors commented that the exposure is not negligible and is associated with an increase in malignancy risk.^20^ Similar data were also recently reported by Voskoboinik et al.^21^ over a 12-year period.

Current recommendations from the American College of Cardiology suggest that all cardiac catheterization laboratories (including electrophysiology laboratories) follow the “as low as reasonably possible” (ALARA) principle.^22^ This concept applies to both patients and medical personnel. For Percell et al.,^1^ with respect to the case presented, the ALARA principle implies the use of no fluoroscopy, even in a complex ablation case requiring a redo procedure. One cannot discern if there is any superiority of one three-dimensional mapping system over the other. However, given the data presented here, I hope that the readers take home the message that ALARA likely means reducing fluoroscopy to an even more significant degree than what we are currently doing, particularly for AF ablation. If such means essentially avoiding all use of fluoroscopy once left atrial access has been achieved, this alone could lead to a significant reduction in X-ray exposure. For those interested in zero-fluoroscopy, I would encourage the review of an excellent “how-to” manuscript by Lerman et al.^23^

## Figures and Tables

**Table 1:** Summary of Studies Employing Zero-fluoroscopy for Atrial Fibrillation

Study	Year Published	Study Design	No. of Patients Treated*	AF Type	Mapping System**	ICE Use?	Energy Type	Procedure Time	Success	Complications
Ferguson et al.^4^	2009	Prospective, observational	21	Paroxysmal + persistent	EnSite™ NavX™	Yes	Irrigated RF	208 (188–221) minutes	100%	0%
Reddy et al.^5^	2010	Prospective, observational	20	Paroxysmal	EnSite™ NavX™	Yes	Irrigated RF	244 ± 75 minutes	97%	0%
Razminia et al.^6^	2014	Retrospective, cohort	5	Paroxysmal	EnSite™ NavX™	Yes	Cryoballoon	181 ± 42 minutes	100%	0%
Percell et al.^7^	2016	Retrospective, cohort	20	Paroxysmal + persistent	CARTO^®^	Yes	Irrigated RF	210.38 minutes	100%	5% (one tamponade)
Bulava et al.^8^	2015	Prospective, randomized	40	Paroxysmal	CARTO^®^	Yes	Irrigated RF	92.5 ± 22.9 minutes	100%	0%
O’Brien et al.^9^	2017	Prospective, observational	55	Paroxysmal + persistent	CARTO^®^	No (TEE)	Irrigated RF	127.71 ± 46.23 minutes	100%	5.4% (three unrelated to the procedure)
Razminia et al.^10^	2017	Retrospective, cohort	186	Paroxysmal + persistent	EnSite™ NavX™ or CARTO^®^	Yes	Irrigated RF or cryoballoon	194.4 minutes	100%	1.6% (two tamponade, one atrioesophageal fistula)
Lyan et al.^11^	2018	Retrospective, cohort	245	Paroxysmal	CARTO^®^	Yes	Irrigated RF	108.8 ± 18.2 minutes	100%	1.2% (three tamponade)

AF: atrial fibrillation; ICE: intracardiac echocardiography; RF: radiofrequency; TEE: transesophageal echocardiography.

*A total of 592 patients were treated.

**The EnSite™ NavX™ system is manufactured by Abbott Laboratories (Chicago, IL, USA), whereas the CARTO^®^ system is manufactured by Biosense Webster (Diamond Bar, CA, USA).
